# Fluoroscopic lumbar transforaminal epidural steroid injections for recurrent herniated intervertebral disc after discectomy: Effectiveness and outcome predictors

**DOI:** 10.1371/journal.pone.0271054

**Published:** 2022-07-07

**Authors:** Mi-Kyung Um, Eugene Lee, Joon Woo Lee, Yusuhn Kang, Joong Mo Ahn, Heung Sik Kang

**Affiliations:** 1 Department of Radiology, Seoul National University Bundang Hospital, Seongnam-si, Republic of Korea; 2 Department of Radiology, Kangwon National University School of Medicine, Chuncheon-si, Republic of Korea; National Institute of Child Health and Human Development (NICHD), NIH, UNITED STATES

## Abstract

**Background:**

Despite transforaminal epidural steroid injection (ESI) being the first choice in patient with recurrent herniated intervertebral disc (HIVD), efficacy of ESI in those patients are not well established. Herein, we evaluate the effectiveness and outcome predictors of fluoroscopic transforaminal ESI for recurrent HIVD.

**Methods:**

Seventy-seven patients (48 male; mean age, 51.3 years) with recurrent lumbar HIVD were included and divided into three groups according to initial treatment: conservative treatment, transforaminal ESI, and immediate surgery. ESI effectiveness was evaluated by operation rates, injection numbers in 6 months, and pain reduction (visual analog scale (VAS) scores). Clinical and MRI variables were analyzed as possible outcome predictors. Each subject in the transforaminal ESI group was individually matched to two patients with initial HIVD (control group).

**Results:**

In the transforaminal ESI group (n = 37), 20 patients (54.1%) did not undergo reoperation. The initial and follow-up VAS scores were significantly higher in the reoperation group (*p = 0*.*014*, *p = 0*.*019*, respectively). Patients with either paresthesia or motor weakness (12/19, 63.2%) had a significantly higher reoperation rate than patients with only pain (5/18, 27.8%; *p = 0*.*031*). Extruded disc ratios ≥2.0 were significantly higher in the reoperation group (10/17, 58.8%; *p = 0*.*048*). The reoperation rate in the transforaminal ESI group (17/37, 45.9%) was higher than the operation rate in the control group (6/73, 8.2%; *p<0*.*001*).

**Conclusion:**

Transforaminal ESI was effective in reducing radicular pain in patients with recurrent HIVD. Approximately 54% of patients did not undergo reoperation. An extruded disc ratio ≥2.0 and paresthesia or motor weakness were poor outcome predictors.

## Introduction

Recurrent lumbar herniated intervertebral disc (HIVD) is defined as the presence of herniated disc material at the previously operated level in a patient who has experienced a pain-free interval since the initial surgery [1]. Despite the availability of improved surgical techniques and instrumentation, recurrent HIVD cannot be avoided. The incidence of recurrent HIVD after discectomy in the lumbar spine ranged from 0% to 23% [2] and recurrent HIVD after discectomy is an important challenge for spine surgeons.

The treatment options for recurrent HIVD include observation, medication, physical therapy for rehabilitation, epidural steroid injection (ESI), or operative intervention. In most cases, medication or ESI is prescribed, and if there is no response or there is motor weakness, the patient will eventually undergo reoperation.

Fluoroscopic ESI is a widely used treatment approach for managing lumbar radicular pain due to HIVD [3]. The efficacy of ESI in patients with HIVD has been demonstrated in several studies [4–7]. Among the various injection methods, transforaminal ESI is the first choice in patients with recurrent HIVD [8]. However, few reports have examined the efficacy of ESI in this group of patients [8–11].

We hypothesized that transforaminal ESI would be effective for radicular pain caused by recurrent lumbar HIVD and that it could also reduce the reoperation rates. Therefore, the aim of this study was to evaluate the effectiveness and outcome predictors of transforaminal ESI for recurrent lumbar HIVD.

## Materials and methods

### Patient selection

This retrospective study was approved by the institutional review board, and the requirement for informed consent was waived. All patients diagnosed with recurrent lumbar HIVD from January 2009 to March 2018 were identified from electronic medical records at our institution.

A total of 123 patients (Male: Female = 45: 79; mean age, 51.8 [range: 23–86] years) who met all inclusion criteria were included in the study. The inclusion criteria were: (1) previous discectomy in one level of the lumbar spine; (2) relapsed lumbar radicular pain with a symptom-free interval after the surgery; (3) evidence of recurrent HIVD at the operated site on follow-up magnetic resonance imaging (MRI); (4) presence of no lesions that could explain the patient’s radicular pain other than recurrent HIVD on follow-up MRI.

The exclusion criteria were: (1) history of ESI at other institutions for radicular pain after discectomy; (2) history of lumbar ESI other than transforaminal ESI, such as interlaminar ESI or caudal injection; (3) the presence of other diseases in the lumbar spine such as metastasis, diskitis, and compression fracture; (4) two or more recurrences after discectomy; and (5) follow-up loss; some patients visited the emergency room or outpatient clinic only once.

**Fig 1:**
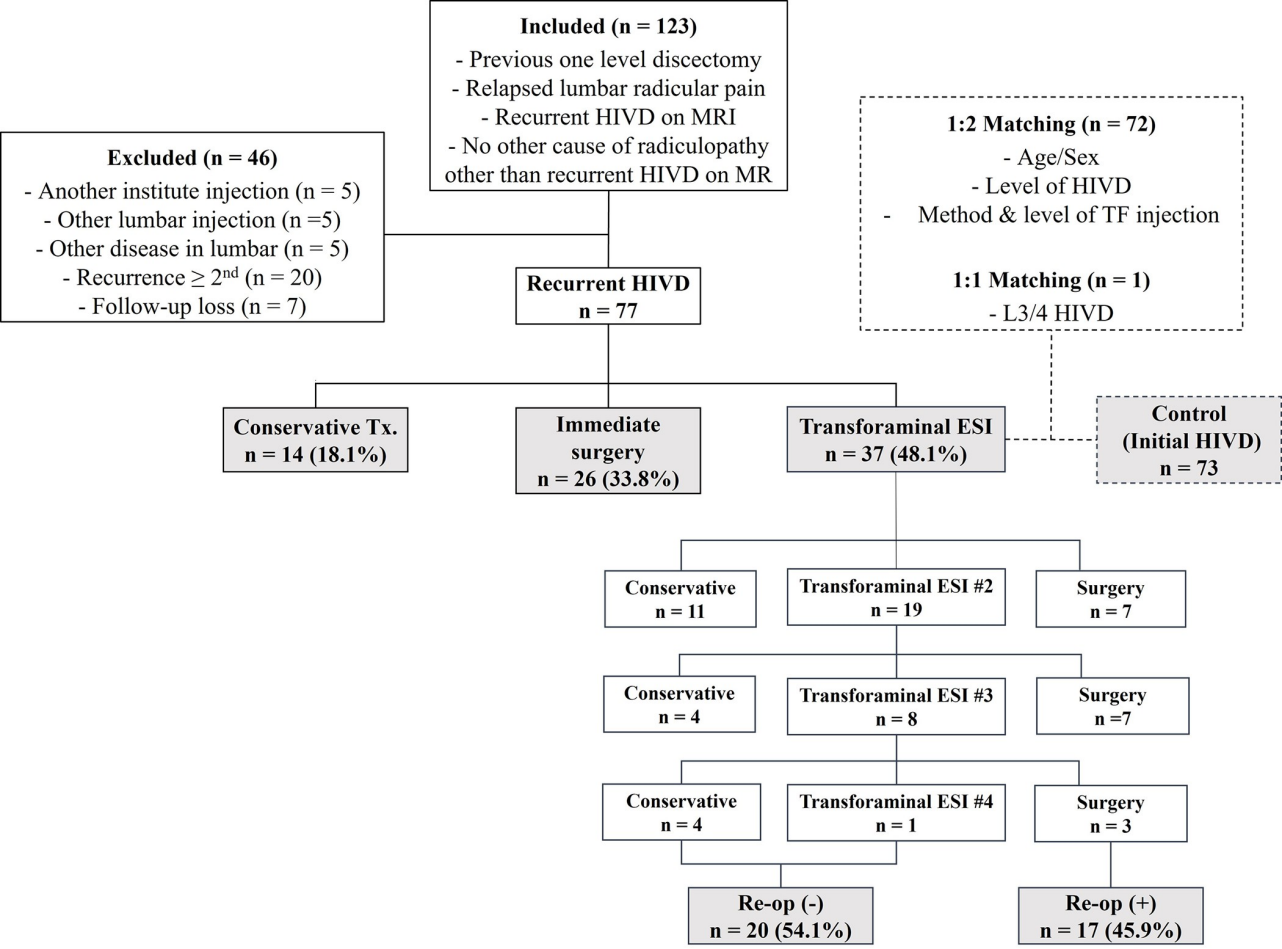
Flow chart for study inclusion/exclusion and follow-up as per initial treatment. Recurrent HIVD patients was dived into three groups according to the treatment. The transforaminal ESI group was compared with initially diagnosed HIVD group (control group). The matching condition with control group was displayed in a dashed box.

Fig 1 displays the flow chart for the study inclusion and exclusion criteria and follow-up as per the initial treatment. A total of 77 patients (Male: Female = 48: 29, mean age, 51.3 [range: 23–86] years) were finally enrolled and were categorized into three groups according to the treatment they received during the initial 6 months after the diagnosis of recurrent HIVD: conservative treatment, immediate surgery group and transforaminal ESI groups.

To examine differences in the effectiveness of transforaminal ESI according to whether or not the operation was performed, patients with initial HIVD who visited the outpatient clinic for spine intervention for lumbar radicular pain and were diagnosed with lumbar HIVD after initial MRI were selected as a matched control group from the cohort population from January 2017 to December 2017. Each patient with recurrent HIVD was individually matched to two separate patients with initial HIVD (at 1: 2 ratio) for age, sex, level of HIVD, and level of ESI. A patient with recurrent HIVD at the L3/4 level was matched with only one patient due to lack of available matched cases.

### Review of clinical data

A retrospective review of the patients’ medical records was performed by one of the authors who was not involved in patient treatment. Information regarding each patient’s age, sex, chief complaint, visual analog scale (VAS) score, pain-free interval, follow-up period, the level of ESI, number of ESIs, and presence of reoperation were obtained by reviewing these records. The pain-free interval refers to the period from symptom relief after surgery to similar or more severe symptom recurrence. The follow-up period was defined as the period between the day of first hospital visit after symptom relapse and the day of the last follow-up in the outpatient clinic. In patients who underwent reoperation, the preoperative follow-up period was defined as the period between the first visit after symptom relapse and the day of the reoperation.

### Magnetic resonance imaging analysis (possible imaging outcome predictors)

MR images were taken with various MR systems from two vendors. Typical acquisition parameters of noncontrast lumbosacral MRI using 3.0T (Ingenia or Achieva, Philips Healthcare) or 1.5T (Amira, Siemens Healthcare) MR systems in our institution are listed in Table 1.

**Table 1:** Typical noncontrast lumbosacral MR acquisition parameters of our institution.

		T1-weighted FSE sequences		T2-weighted FSE sequences	
		Transverse	Sagittal	Transverse	Sagittal
**3T**	TR (ms)	450–600	400–600	4000–6300	2000–3700
	TE (ms)	9–12	9–10	100–120	120
	Matrix size	256 x 240–256	350–900 x 250–350	256 x 240–256	350–900 x 250–300
	FOV (cm)	150 x 150	400–610 x 300–350	150 x 150	400–610 x 300–350
	Section thickness (mm)	4	4	4	4
	Echo-train length	4–7	5–7	24–30	20–30
	No. of acquisitions	1–2	1–2	1–2	1–2
**1.5T**	TR (ms)	400	600	2350	4220
	TE (ms)	11	8.2	88	129
	Matrix size	320 x 320	512 x 512	320 x 320	512 x 512
	FOV (cm)	180 x 180	340 x 340	180 x 180	340 x 340
	Section thickness (mm)	4	4	4	4
	Echo-train length	3–5	6–7	16–17	13–20
	No. of acquisitions	1–2	1–2	1–2	1–2

FSE indicates fast spin echo; TR, repetitive time; TE, echo time; FOV, field of view; No., number.

**Fig 2:**
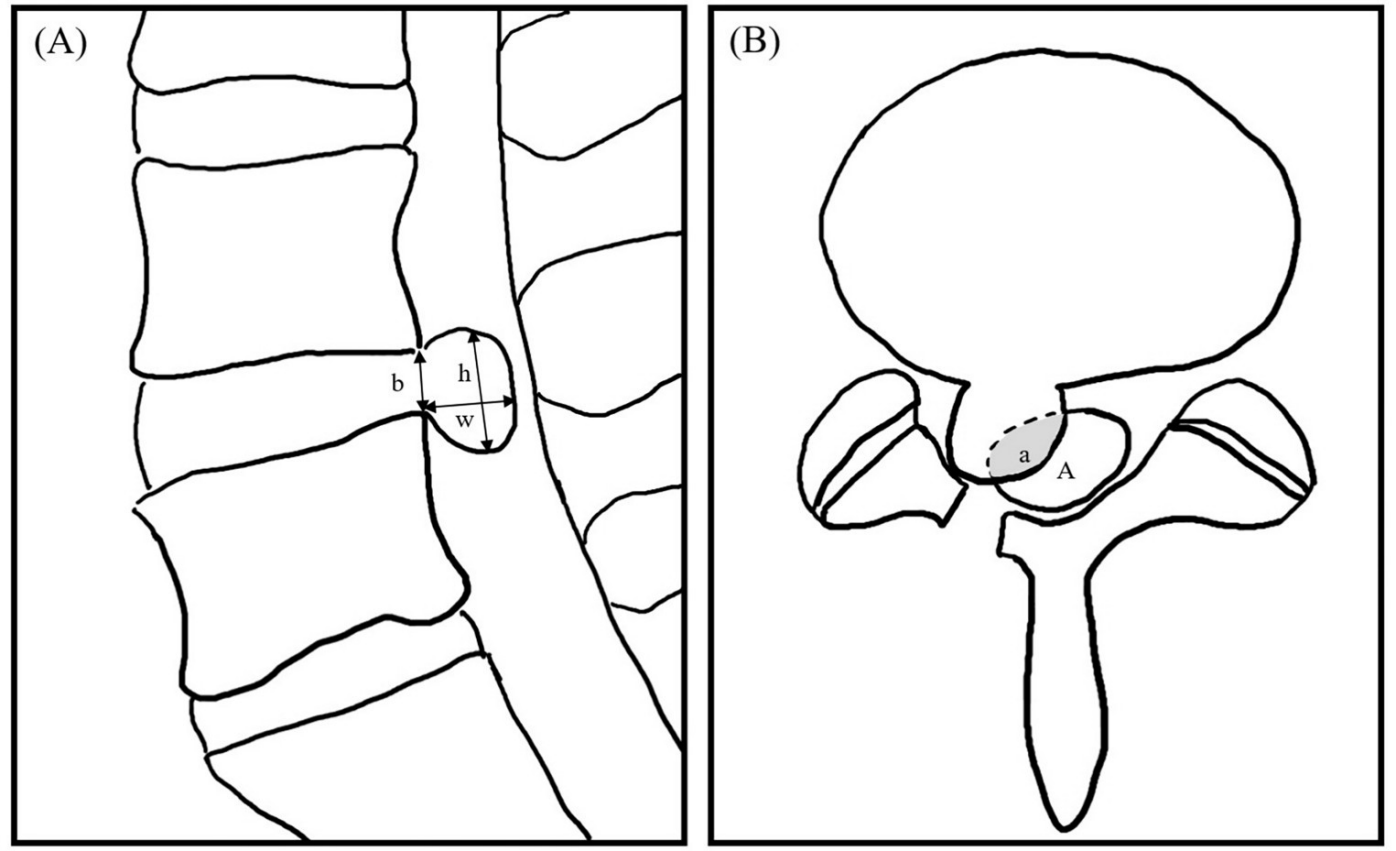
Schematic diagrams of the disc ratio and percentage of central canal compromise. (A) The ratio was obtained at the sagittal scan where the extruded disc size is greatest. The ratio was calculated by dividing the larger value of the width (w) or the height (h) by the length of the base [ratio = (larger value of w or h) /b]. (B) The percentage of central canal compromise was obtained from the axial scan where the extruded disc size is greatest. The percentage of central canal compromise was calculated by dividing the expected area of central canal compromise by the extruded disc by the expected area of the central canal [a/A x 100(%)]. b: base of extruded disc, h: height of extruded disc, w: anteroposterior width of extruded disc, a (grey area): expected area of central canal compromise by extruded disc, A: expected area of the central canal.

**Fig 3:**
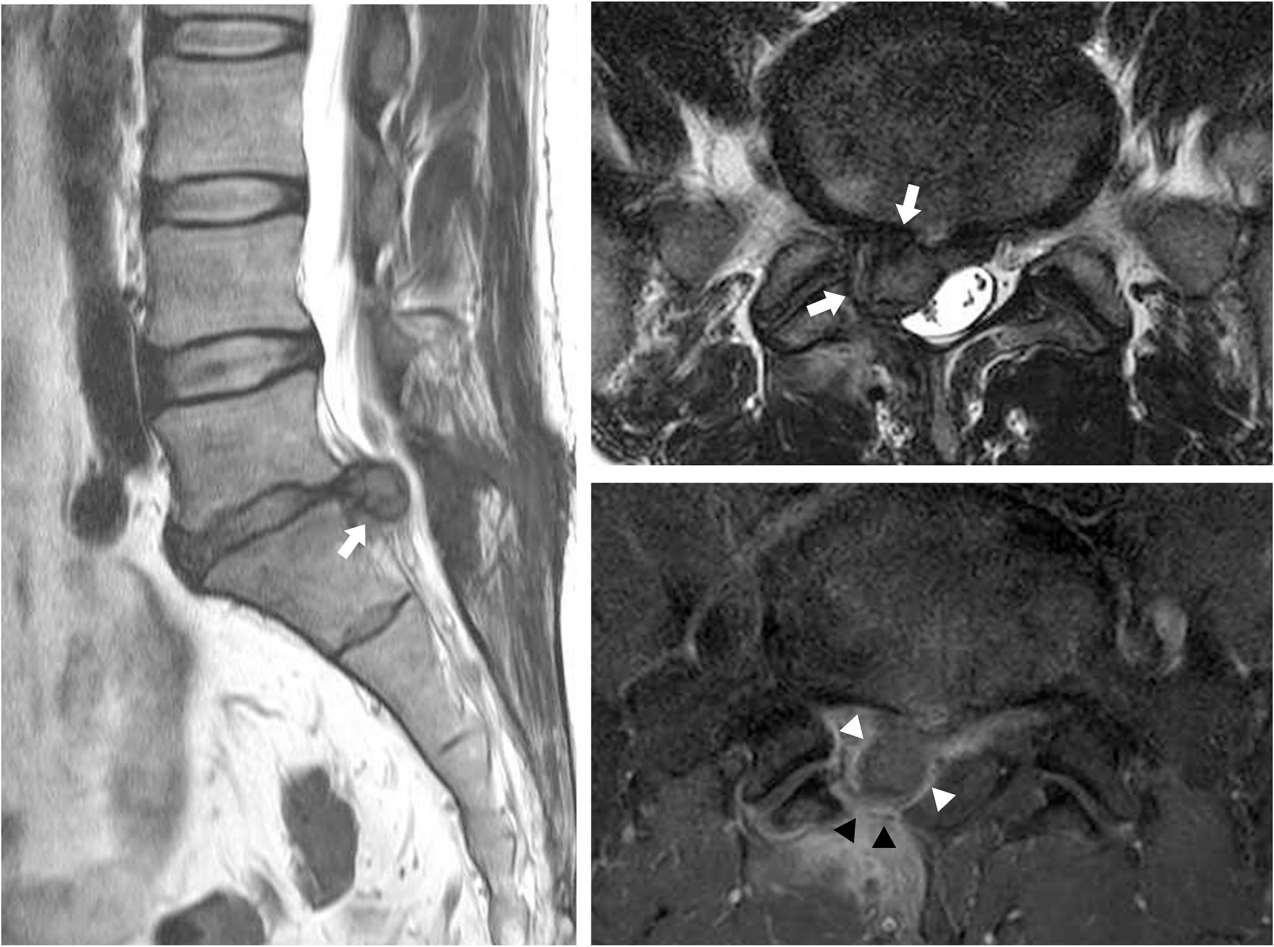
Magnetic resonance imaging of a 41-year-old man with radiating pain to the left buttock and lower leg in the S1 dermatome. T2-weighted sagittal and axial images show recurrent herniated intervertebral disc (white arrows) in the right central zone at the level of L5/S1 with inferior migration causing compression of the right S1 root. The extruded disc (white arrow heads) shows rim-enhancement on the contrast-enhanced T1-weighted axial scan. Diffuse contrast enhancement without mass effect to the spinal canal at the right partial hemilaminectomy site (black arrow heads) of L5 indicating postoperative changes.

Two radiologists with experience of 12 and 5 years, respectively, who were blinded to the clinical data, reviewed only the MR images in consensus for the following items: (1) level of HIVD; (2) zone of HIVD (central, subarticular, and foraminal); (3) migration of the disc (no, inferior, or superior); (4) relation of HIVD to the nerve root (no contact, contact with, or compression of the nerve root), (5) ratio of the extruded disc (Fig 2A), and (6) percentage of the central canal compromise (Fig 2B). The ratio of the extruded disc was measured by the ratio of the base and the maximal distance between the edges of the disc material in the sagittal scan where the distance between the edges of the extruded disc was greatest. The ratio of the extruded disc was calculated by dividing the larger of the width or the height by the length of the base. The ratios were divided into two groups: lower than 2.0 and equal to or higher than 2.0. The percentage of the central canal compromise was obtained by dividing the expected area of central canal compromise by the extruded disc by the area of the central canal after measuring the cross-sectional area of the central canal and the area of the disc extruding into the central canal. The percentages of central canal compromise were classified into two groups: lower than 25% and equal to or higher than 25%. Fig 3 shows typical MRI findings of recurrent HIVD.

### Evaluation of effectiveness and possible outcome predictors

The effectiveness of transforaminal ESI was evaluated by the operation rates, number of injections in 6 months, and pain reduction using the VAS score. Possible clinical and imaging outcome predictors were determined. The assessed clinical predictors were patient age and sex, chief complaint (pain, paresthesia, or motor weakness), and VAS score (initial, 2-week follow-up, and difference). The VAS score difference was calculated by subtracting the follow-up VAS score from the initial VAS score. The possible imaging outcome predictors were the same as the parameters described in the MRI analysis.

### Transforaminal ESI method

All lumbar transforaminal ESIs were performed by one of two radiologists with experience in spinal intervention of 12 and 7 years, respectively. Injections were performed at the level that best matched the patient’s clinical symptoms. The level of the transforaminal ESI was determined according to the migration of the disc. The injection was performed at the same level of HIVD if there was no inferior migration of the disc and at one level below if there was inferior migration of the disc.

**Fig 4:**
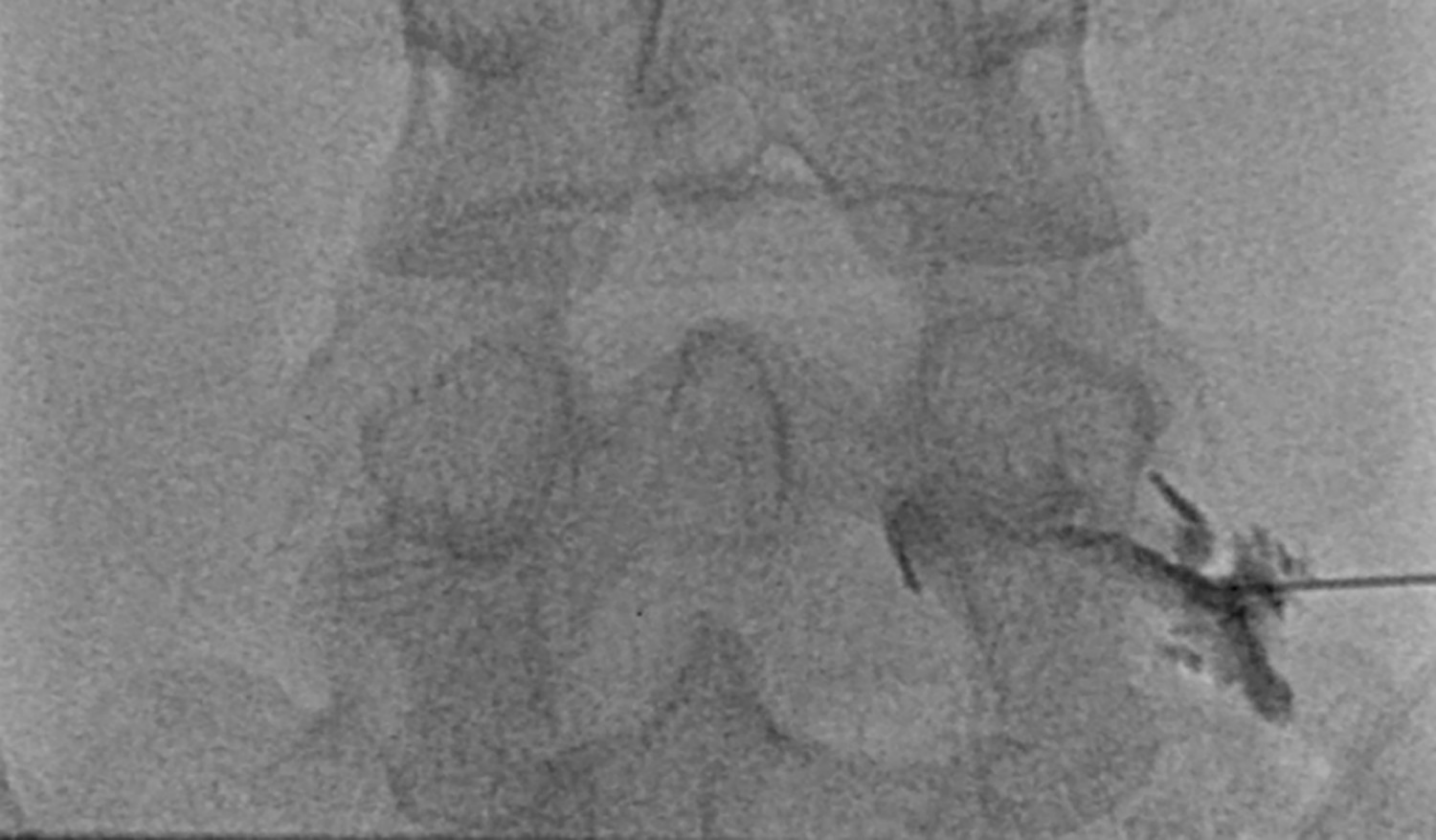
A spot radiograph of a 38-year-old man with radiating pain to the right buttock and lower leg in the L5 dermatome. A magnetic resonance image (not shown) revealed a right central L5-S1 herniated intervertebral disc with compression of the right S1 root. A transforaminal injection was performed at the L5-S1 neural foramen.

Patients were placed in the prone position on a fluoroscopy table. Under fluoroscopic guidance, a 22G spinal needle was advanced *en face* from the skin to the epidural space through the neural foramen for the transforaminal ESI with the conventional or the posterolateral technique [12]. After the needle position was inspected, approximately 1 mL of contrast media, iohexol with 300 mg of iodine per mL (OMNIPAQUE 300^Ⓡ^; Amersham Health, Princeton, NJ), was injected to confirm the precise epidural location of the needle tip. Ten milligrams (2 mL) of dexamethasone (5 mg/mL) was injected, and then an admixture of 3.75 mg (0.5 mL) of ropivacaine hydrochloride (7.5mg/mL) and 1 mL of saline was administered [13]. Fig 4 shows an example of a fluoroscopy-guided transforaminal ESI at the L5-S1 level.

### Statistical analysis

For statistical analysis, the transforaminal ESI group was sub-divided into two groups according to the reoperation status. For these two groups, age and VAS scores were evaluated using t-tests. Sex, chief complaints, the level and zone of HIVD, injection number, ratio of the extruded disc, and the percentage of central canal compromise were evaluated using the chi-square test. Presence of root compression was evaluated using Fisher’s exact test. One patient in the non-reoperation group had HIVD at the L3/4 level, and this patient was excluded from the disc-level statistical analysis due to the absence of statistical significance. A patient with sequestered disc and three patients with herniated disc at the foraminal zone were excluded when comparing the area of central canal compromise.

The transforaminal ESI group was compared with the matched control group, i.e., with those who were initially diagnosed with HIVD. For the two groups, the VAS score and the average number of injections in 6 months were evaluated using t-tests. Chief complaints and operation rates were evaluated using the chi-square test. All statistical analyses were performed with statistical software (SPSS 23; SPSS, Chicago, IL). A *p*-value lower than *0*.*05* was considered to indicate a significant difference.

## Results

### Comparison among the groups of operated patients

The patients with recurrent HIVD (n = 77) were divided into three groups according to the initial treatment: (1) the conservative treatment group (n = 14, 18.1%), (2) the transforaminal ESI group (n = 37, 48.1%), and (3) the immediate surgery group (n = 26, 33.8%).

In the conservative treatment group, the mean pain-free interval was 1458.1 days [range, 30–4380 days] with a mean follow-up period of 440.4 days [range, 7–2829 days]. Of the 14 patients, three were observed without special medication, eight were prescribed nonsteroidal anti-inflammatory drugs and muscle relaxants, and an oral steroid was prescribed in one patient. Two patients were prescribed rehabilitation exercise. Most of the patients in the conservative group had no recorded VAS score and only minor symptoms were described in the medical records. None of the patients in this group underwent reoperation or received epidural steroid injection until the final follow-up day.

The mean pain-free interval of the immediate surgery group was 664.3 days [range, 7–4015 days] and the mean follow-up period was 1334.7 days [range, 35–3258 days]. The immediate surgery group underwent reoperation after a mean follow-up period of 10.4 days [range, 2–42 days], which is a period between the first visit after symptom relapse and the day of the reoperation. Reoperation was determined by the surgeon taking into account pain severity, motor weakness, paresthesia, and many other conditions. Reoperation was first considered in patients with severe pain recurrence during hospitalization after discectomy or in patients with severe motor weakness. There was symptom alleviation after initial surgery in three patients, and during their first hospital stay, they complained of pain similar to or worse than that before surgery. Five patients complained of relapsed symptom at the follow-up clinic in a month after the first discectomy. Another five patients complained of recurred radicular pain within 6 months from the first discectomy. The rest 13 patients relapsed pain and revisited outpatient clinic after 6 months from the initial discectomy. MRI scans revealed recurrent HIVD, and they underwent reoperation.

The mean pain-free interval in the transforaminal ESI group was 1315.5 days [range, 30–6935 days]. The mean follow-up period in the transforaminal ESI group was 10187.1 days [range, 3–332256 days]. Thirty-seven patients in the transforaminal ESI group received a total of 64 injections. The average number of transforaminal ESIs was 1.73 (± 0.80). None of the patients experienced major complications after ESI that required hospitalization or visit to the emergency room [14].

The initial VAS scores of the transforaminal ESI and immediate surgery groups were 7.52 and 7.50, respectively, and they were not significantly different (*p = 0*.*975*). In the immediate surgery group, more patients complained of motor weakness (9/26, 34.5%) compared to the transforaminal ESI group (7/37, 18.9%), but there was no significant difference between the two groups (*p = 0*.*240*). Paresthesia did not significantly differ between the two groups. In the transforaminal ESI group, 17 (45.9%) patients underwent reoperation. Finally, of the 77 patients with recurrent HIVD, 43 (55.8%) underwent reoperations.

The demographics and clinical variables of the transforaminal ESI group are presented in Table 2. The results were compared by dividing the patients into two subgroups according to the reoperation status. Patients with either paresthesia or motor weakness (12/17, 70.6%) showed a significantly higher reoperation rate after ESIs than that in patients with only pain (5/17, 29.4%; *p = 0*.*031*). The initial VAS and the follow-up VAS scores were significantly higher in the reoperation group (*p = 0*.*014*, *p = 0*.*019*, respectively). The VAS score difference was higher in the non-reoperation group than in the reoperation group; however, these differences were not significant (*p = 0*.339). Other clinical variables were not statistically significant outcome predictors.

**Table 2:** Clinical findings related to patient outcome in the transforaminal ESI group.

	Non-reoperation group n = 20 (54.1%)	Reoperation group n = 17 (45.9%)	*p-value*
**Age, mean (std)**	50.4 (15.7)	50.5 (19.0)	*0*.*983*
**Sex**			*0*.*157*
M	14 (70.0)	8 (47.1)	
F	6 (30.0)	9 (52.9)	
**Chief complaint**			
Pain	20 (100)	17 (100)	
Pain and paresthesia	7 (35.0)	11 (64.7)	
Pain and motor weakness	3 (15.0)	4 (23.5)	
All	3 (15.0)	3 (17.6)	
**Chief complaint (dichotomous analysis)**			***0*.*031***
Pain only	13 (65.0)	5 (29.4)	
Pain with either paresthesia or motor weakness	7 (35.0)	12 (70.6)	
**Visual Analog Scale (VAS)**			
Initial	6.6	8.4	***0*.*014***
2week F/U	3.7	6.0	***0*.*019***
Difference	3.4	2.4	*0*.*339*
**Injection number**			*0*.*372*
1	11 (55.0)	7 (41.2)	
2	4 (20.0)	7 (41.2)	
3	5 (25.0)	3 (17.6)	
Average (std)	1.70 (0.25)	1.76 (0.25)	*0*.*811*

HIVD, herniated intervertebral disc; ESI, epidural steroid injection; std, standard deviation.

Values inside parentheses indicate percentages, except for age and average injection number.

Value inside parentheses for age and average injection number indicate standard deviation.

Table 3 shows the MRI findings related to patient outcomes in the transforaminal ESI group. A ratio of extruded disc equal or higher than 2.0 was significantly higher in the reoperation group (10/17, 58.8%, *p = 0*.*048*). Other imaging parameters were not significantly different.

**Table 3:** MRI findings related to patient outcome in the transforaminal ESI group.

	Re-op (-) n = 20 (54.1%)	Re-op (+) n = 17 (45.9%)	*p-value*
**Disc herniation level** *			*0*.*985*
L4/5	9 (45.0)	8 (47.1)	
L5/S1	10 (50.0)	9 (52.9)	
**Disc location**			*0*.*942*
Central	12 (60.0)	10 (58.8)	
Subarticular and foraminal	8 (40.0)	7 (41.2)	
**Disc migration**			*0*.*315*
Migration (-)	6 (30.0)	3 (17.6)	
Migration (+)	14 (70.0)	14 (82.4)	
**Root compression**			*0*.*715*
Compression (-)	1 (5.0)	1 (5.9)	
Compression (+)	19 (95.0)	16 (94.1)	
**Ratio of disc base and maximal distance between edges** **			***0*.*048***
1 < ratio < 2	14 (73.7)	7 (41.2)	
≥ 2.0	5 (26.3)	10 (58.8)	
**Area of central canal compromise** ***			*0*.*579*
< 25%	9 (52.9)	10 (62.5)	
≥ 25%	8 (47.1)	6 (37.5)	

MRI, magnetic resonance imaging; HIVD, herniated intervertebral disc; ESI, epidural steroid injection; std, standard deviation.

*A patient with recurrent HIVD at L3/4 was excluded for statistical analysis when comparing disc level

** A patient with sequestered disc was excluded when comparing the ratio of disc base and maximal distance between edges.

*** A patient with sequestered disc and three patients with herniated disc at foraminal zone were excluded when comparing area of central canal compromise.

### Comparison between operated and non-operated patients

In order to compare the effectiveness of ESI, the patients in the transforaminal ESI group were individually matched with the patients in the initial HIVD group (control group). The mean follow-up period in the control group was 201.1 days [range, 2–679 days]. Table 4 shows the demographics and clinical variables of the transforaminal ESI and control groups. The initial and follow-up VAS scores and the VAS score difference were not significantly different between the two groups (*p = 0*.*944*, *p = 0*.*943*, *p = 0*.*363*, respectively). The reoperation rate in the transforaminal ESI group (17/37, 45.9%) was significantly higher than the operation rate in the control group (6/67, 8.2%; *p < 0*.*001*).

**Table 4:** Clinical findings related to patient outcome after lumbar transforaminal ESI between lumbar transforaminal ESI group and control group.

	Transforaminal ESI n = 37	Control n = 73	*p-value*
**Age, mean (std)**	50.4 (16.6)	50.2 (16.8)	*0*.*944*
**Sex**			*0*.*614*
M	22 (59.5)	47 (64.4)	
F	15 (40.5)	26 (35.6)	
**Chief complaint (dichotomous analysis)**			*0*.*403*
Pain only	18 (48.6)	29 (39.7)	
Pain with either paresthesia or motor weakness	19 (51.4)	43 (58.9)	
**Visual Analog Scale (VAS)**			
Initial	7.5	7.6	*0*.*944*
2week F/U	4.9	5.5	*0*.*943*
Difference	2.5	2.1	*0*.*363*
**Injection number**			*0*.*120*
1	19 (51.4)	40 (54.8)	
2	10 (27.0)	27 (37.0)	
3	8 (21.6)	6 (8.2)	
Average (std)	1.70 (0.81)	1.52 (0.67)	*0*.*253*
**Operation rate** *			***0*.*000***
Operation (-)	20 (54.1)	67 (91.8)	
Operation (+)	17 (45.9)	6 (8.2)	

HIVD, herniated intervertebral disc; std, standard deviation.

Value inside parentheses for age, VAS and average injection number indicate standard deviation

* Operation rate for transforaminal ESI group indicates reoperation rate.

## Discussion

This study revealed important information regarding the effectiveness and outcome predictors of transforaminal ESI for recurrent lumbar HIVD. Our findings demonstrated that similar to that for initial HIVD, transforaminal ESI for recurrent HIVD effectively reduces radicular pain. Approximately 54% of patients with recurrent HIVD did not undergo reoperation after lumbar transforaminal ESI. Paresthesia or motor weakness was a poor outcome predictor. A ratio of extruded disc of ≥2.0 was found to be a poor imaging outcome predictor. Few studies have previously examined the efficacy of ESI for recurrent HIVD [8–11], and there is no consensus on how physicians should manage patients with recurrent lumbar HIVD.

The reported incidences of recurrent lumbar HIVD vary between 3% and 24%, reflecting the variability in surgical techniques, follow-up, and management paradigms among investigations [15–18]. According to a review by Parker et al. [2], the cumulative incidence of recurrent HIVD was 5.3% and the rate of reoperation was 4.4%. According to Kim et al. [18], the cumulative reoperation rate after surgery for lumbar HIVD was 13.4% after 5 years. Although the incidence of recurrent disc herniation was lower than that of delayed postoperative back pain in the study by Parker et al. [2], its severity was markedly greater, and patients required reoperation in most cases.

In this study, approximately 54% of the patients in the transforaminal ESI group did not undergo reoperation. These results suggest that transforaminal ESI may be an effective treatment in patients with recurrent lumbar HIVD. Several factors may have resulted in approximately half the patients with transforaminal ESI avoiding reoperation. Transforaminal ESI in patients with recurrent HIVD can result in effective pain relief, as observed in patients with HIVD who have not undergone surgery. In our study, there was no significant statistical difference in the initial and follow-up VAS scores and in the VAS score difference and the average injection number in 6 months between the transforaminal ESI and control groups. These results suggest that these two groups may show similar clinical features and transforaminal ESI treatment outcomes regardless of whether they undergo discectomy. In our study, the initial and follow-up VAS scores after initial injection were significantly higher in the reoperation group than in the non-reoperation group. This could be the reason for the high reoperation rate. A VAS difference of ≥3 points, signifying a reduction in pain of at least 30%, is considered a clinically significant result, as reported by previous studies [19, 20]. The non-reoperation group had pain reduction higher than 30%; however, the reoperation group had pain reduction lower than 30%, which was not sufficient according to the aforementioned criterion. This indicates that transforaminal ESI was less effective in the reoperation group.

One clinical difference is that there was a higher rate of reoperation in the transforaminal ESI group than in the control group or than that reported in other studies [2, 18]. In our routine clinical process, patients requiring transforaminal ESI were referred by physicians, usually neurosurgeons and orthopedic surgeons. Patients who did not respond well to conservative treatments in their outpatient clinic were referred for spinal injections. In addition, patients with a history of previous ESI or follow-up loss were excluded in this study, who may have shown relatively good prognosis. Therefore, it is possible that the proportion of patients with symptoms severe enough to consider reoperation was high due to the study design. This can also be inferred by fact that most patients in our study who performed conservative treatment according to symptom improvement over time or those who rejected transforaminal ESI also showed a high follow-up loss rate. Our results suggest that in half the cases wherein symptoms were severe enough to consider interventional or surgical treatment, these improved with transforaminal ESI; reoperation was required in the remaining cases. Although it cannot be concluded that transforaminal ESI lowers the reoperation rate in patients with recurrent HIVD after discectomy, these findings may suggest that treatment with transforaminal ESI can be attempted before determining the need for reoperation. Additional comparative investigations are required to confirm whether transforaminal ESI actually reduces the reoperation rate. The following are some of the reasons why the reoperation rate was so high in patients with recurrent HIVD. Reoperation rates are likely to be influenced by patient factors, and these factors affect physician decisions. In fact, there were no statistical differences in the initial VAS scores between the transforaminal ESI and control groups, and there was no significant difference in the degree of pain relief. This signifies that compared to patients with initial HIVD, patients with recurrent HIVD with a high initial VAS score are more likely to undergo reoperation instead of nonsurgical management. Patients with recurrent HIVD are possibly more sensitive to pain because they have previously experienced radicular pain. In this study, 43 of 77 patients underwent repeated discectomies, and all patients underwent reoperation due to severe pain complaints according to the medical records. Moreover, some patients consider recurrent HIVD as surgery failure. This notion is burdensome to surgeons and can result in quick decisions for reoperation by patients and physicians. In our study, patients in the immediate surgery group underwent reoperation only mean 10.4 days after diagnosis. Considering that the absolute indication of discectomy for HIVD in South Korea is cauda equina syndrome, there was no significant statistical difference in motor weakness between the transforaminal ESI and control groups, and it is not possible to conclude that patients with recurrent HIVD are more likely to have motor weakness.

Among the clinical predictive factors, ESI showed efficacy in pain reduction; however, it was less effective in patients with paresthesia or motor weakness. These results are consistent with those of prior studies on ESI in patients with lumbar radicular pain due to HIVD [4, 21, 22]. In a previous study by Choi et al. [23], the location of HIVD and grade of nerve root compression were possible outcome predictors for ESI. The study by Lee et al. [24] reported that HIVD in the foraminal or extraforaminal zone was the MRI-based outcome predictor for lumbar transforaminal ESI. In this study, the incidence of an extruded disc ratio of ≥2.0 was significantly higher in the reoperation group. However, other imaging factors were not significant in our study, including root compression, location of the disc, and percentage of central canal compromise. The reason why other factors were not meaningful in this study could be the decompressed state of the central canal from the previous laminectomy procedure performed with the discectomy. In patients who undergo discectomy, the ratio of the extruded disc on the sagittal scan could be an imaging outcome predictor. The ratio of the extruded disc was measured in both sagittal and axial scans; however, no patient had a larger ratio in the axial scan. It is possible that patients who undergo discectomy show a relatively broad base on the axial scan.

As with all studies, this study also had limitations. First, it was retrospective, and therefore, there were some data losses resulting from incomplete data collection. The VAS scores of the patients in the conservative group were not recorded, and only mild symptoms were described in the medical records. Although most patients in the conservative group showed improvement with medication, we could not compare the results among the conservative, transforaminal ESI, and immediate surgery groups. Second, the protocol for recurrent HIVD treatment was not standardized. However, as all injections were transforaminal ESIs and matched using the same method, the lack of standardization probably did not confound the analysis of the results for transforaminal ESI. Additional research for other types of ESI, such as interlaminar or caudal, is required. The third limitation is that only the 2-week follow-up VAS scores were available. Other clinical metrics of pain, disability, quality of life, or long-term VAS scores (such as those at 4 or 6 weeks) were not assessed. This is a retrospective study, so variable clinical metrics or long-term follow-up were limited. Moreover, an abundant body of literature shows that typically, pain relief from lumbar transforaminal ESI lasts for 3 to 6 months. However, this varies significantly between patients. Therefore, large cohort studies with multivariate analyses in patients with recurrent HIVD are necessary to investigate the long term effect and outcome predictors of transforaminal ESI. The fourth limitation is the relatively small number of patients. The lack of statistically significant differences despite higher pain reduction rate in patients who did not undergo surgery was probably due to the insufficient number of patients. This study was a retrospective study, and retrospective selection of the participants is predisposed to a high level of bias. We reviewed a decade’s worth of data and found hundreds of patients diagnosed with recurrent HIVD in one database. However, many patients did not have postoperative MRI to prove recurrent HIVD. These patients possibly showed minor symptoms and symptom relief in a short period of time. We may have excluded several patients with recurrent HIVD who had minor symptoms. Further, we also excluded 46 patients according to the exclusion criteria, including those who underwent repeated discectomies. Patients who underwent repeated surgeries had more severe adhesions and architectural distortion compared with those who underwent discectomy only once. Moreover, in the second or third operation, many patients underwent posterior fusion in addition to discectomy; we believed that this would bias the effects of transforaminal ESI and excluded such cases. However, this exclusion could have led to a high level of bias. We also compared the transforaminal ESI group with the control group to examine differences in the effectiveness of transforaminal ESI, but the number of control groups was insufficient in terms of statistical power. In order to maximize the statistical power, we tried to match as many control participants as possible, but the cohort population could only be matched with a 1:2 ratio within the relevant study period. A matching ratio of 1:3 or more could not be obtained due to the limited number of cases, as we matched patients based on age, sex, level of HIVD, and level of ESI. Further evaluation with a larger number of patients to is necessary to validate our findings, including rigorous prospective studies of outcome predictors after ESI to collect further patient information. We hope that our study will form the basis for further prospective studies on the effectiveness of ESI in patients with recurrent HIVD.

In conclusion, transforaminal ESI was effective in reducing radicular pain in patients with recurrent HIVD. Approximately 54% of the patients did not undergo reoperation, and paresthesia or motor weakness were poor outcome predictors. Ratio of the extruded disc of ≥2.0 was a poor imaging outcome predictor. Therefore, transforaminal ESI could be considered before repeat discectomy in patients with recurrent HIVD.

## Supporting information

S1 Data (XLSX)
